# Adipose-derived mesenchymal stem cells attenuate ischemic brain injuries in rats by modulating miR-21-3p/MAT2B signaling transduction

**DOI:** 10.3325/cmj.2019.60.439

**Published:** 2019-10

**Authors:** Chunshuang Li, Kexin Fei, Feipeng Tian, Changkui Gao, Yang Song

**Affiliations:** 1 Department of Emergency, Longnan Hospital of Daqing, Daqing, China; 2 Department of Bone Surgery, Longnan Hospital of Daqing, Daqing, China; The first two authors contributed equally.

## Abstract

**Aim:**

To explore the mechanism underlying the protective effect of adipose-derived mesenchymal stem cells (ADMSCs) against ischemic stroke by focusing on miR-21-3p/MAT2B axis.

**Methods:**

Ischemic brain injury was induced in 126 rats by middle cerebral artery occlusion (MCAO). The effect of ADMSC administration on blood-brain barrier (BBB) condition, apoptosis, inflammation, and the activity of miR-21-3p/MAT2B axis was assessed. The role of miR-21-3p inhibition in the function of ADMSCs was further validated in *in vitro* neural cells.

**Results:**

ADMSCs administration improved BBB condition, inhibited apoptosis, and suppressed inflammation. It also reduced the abnormally high level of miR-21-3p in MCAO rats. Dual luciferase assays showed that miR-21-3p directly inhibited the MAT2B expression in neural cells, and miR-21-3p inhibition by inhibitor or ADMSC-derived exosomes in neurons attenuated hypoxia/reoxygenation-induced impairments similarly to that of ADMSCs *in vivo*.

**Conclusion:**

This study confirmed the protective effect of ADMSCs against ischemic brain injury exerted by suppressing miR-21-3p level and up-regulating MAT2B level.

Cerebral damage due to ischemic stroke (IS) ranks third among mortality-related factors in developed countries and substantially contributes to disability rates worldwide (1,2). IS kills numerous neurons in the central neuron system, and the affected tissue rarely functionally recovers (3). The most effective treatment strategy for IS is thrombolytics administration. However, the effectiveness of this strategy is limited by the narrow time window for its application and hemorrhage complications (4,5), stressing the need for novel effective approaches with few side effects.

Sequencing techniques enabled the discovery of intrinsic factors that regulate neuron survival during IS (6,7). One of the potential treatment targets are microRNAs (miRs), whose expression and function are influenced by IS (8). In an animal model, ischemic injuries affected the expressions of 114 miRs (9). Also, miR-21 and miR-29b levels responded to oxygen-glucose deprivation in cultured neurons and astrocytes (10). Moreover, miR-21-3p level suppression by human umbilical vein endothelial cell-derived exosomes attenuated hypoxia/reoxygenation (H/R)-induced apoptosis in neural cells (11), further confirming miR-21 involvement in the progression of IS injuries. IS injuries induce the release of cytokines, chemokines, and adhesion molecules, which influences the permeability of the blood-brain barrier (BBB) (12,13), promoting the traffic of leukocytes into brain tissues and exacerbating ischemic injuries (12). The increased miR-21-3p level aggravates BBB damage by promoting cellular apoptosis and inflammation by modulating MAT2B function (14). Therefore, miR-21-3p inhibition by a specific agent might be a novel strategy to improve BBB condition in a multi-pronged IS attack.

Cytotherapy is a promising option for the treatment of IS-related disorders, with experimentally and clinically proved efficacy (15-19). Stem cell-based therapies that modulate miRs expression showed considerable feasibility and safety (15). For example, bone marrow mesenchymal stem cells (BMSCs) have shown promise in the treatment of IS injuries (20,21). However, their application is limited due to the invasive methods needed to obtain these cells. Compared with BMSCs, adipose-derived mesenchymal stem cells (ADMSCs) are an abundant stem cell type easy to obtain without ethical issues (22). Chi et al (23) confirmed that ADMSCs alleviated inflammatory response associated with IS and improved BBB condition by suppressing endoplasmic reticulum stress. Moreover, the alleviating effect of ADMSCs on inflammatory response in a liver injury model was reported to be stronger than that of BMSCs (22). Thus, understanding the mechanism behind the anti-IS effect of ADMSCs can facilitate the clinical application of these cells. The aim of the study was to assess the effect of ADMSCs administration on the inflammatory response, BBB permeability, and the activity of miR-21-3p/MAT2B axis.

## Methods

### Adipose-derived mesenchymal stem cells (ADMSCs) identification

ADMSCs were provided by Jilin University (Changchun, China) and were cultured in DMEM (SH30023.01B, Hylone, Carlsbad, CA, USA) in an atmosphere consisting of 5% CO_2_ at 37°C. The stem-cell features of ADMSCs were identified by assessing the osteogenic and adipogenic differentiation potential with Alizarin Red S and Oil Red O experiments, respectively, as previously described (24) (Supplementary Figure 1(supplementary Figure 1)). Moreover, 30 minutes before injection, the surface levels of CD29, CD34, CD44, CD45, and CD90 were detected with immunofluorescent assays (Supplementary Table 1(supplementary Figure 1)) (Supplementary Figure 1). Exosomes were also collected from ADMSCs ((supplementary Figure 2)Supplementary Figure 2).

### Induction of ischemic injuries and ADMSC administration

Male adult Sprague-Dawley rats (weighing 200-300 g) were obtained from Changsheng Biotechnology Inc. (Benxi, Liaoning, China) and housed at room temperature (20-25°C) with a constant humidity (55 ± 5%) and access to food and water. All the animal experiments were conducted in accordance with the Institutional Animal Ethics Committee and Animal Care Guidelines of Longnan Hospital of Daqing and approved by the hospital’s Ethics Committee in 2016. The ischemic injuries were induced using MCAO: briefly, left common carotid artery was exposed and a small incision was made. The left middle cerebral artery was occluded with a 0.28-mm nylon filament for one hour and scars were closed. To verify the effect of ADMSCs, 126 animals were randomly divided into three groups (42 in each group) with use of random number method (mice with No. from 1-42, 43-84, and 85-126 were classified into sham, MCAO, and MCAO+ADMSC groups, respectively): sham group – animals were subjected to standard MCAO without ligation of nylon filament; MCAO group – animals underwent MCAO; MCAO+ADMSC group – rats received tail vein injection of 2.0 × 10^6^/0.5 mL ADMSCs three times at 0, 12, and 24 hours after MCAO (22,23). Of the 42 rats in each group, 18 were used in BBB permeability assay (six for each recording point), six in TUNEL staining, six in TTC staining, six in immunofluorescent assay and ELISA assays, and the remaining six in reverse transcription real time polymerase chain reaction (RT^2^-PCR) and Western blotting assays.

### BBB permeability assay

BBB permeability was measured using Evans Blue dye method 0, 7, and 14 days after MCAO. Briefly, 0.2 mL Evans Blue dye (2%, m/v) (314-13-6, Sigma, St Louis, MO, USA) was intravenously injected in the tail vein five hours before rats were sacrificed. The brain tissue was homogenized and centrifuged at 15 000 × g for 30 min. After being mixed with the same volume of 50% trichloroacetic acid and incubated at 4°C overnight, the mixture was centrifuged at 15 000 g for 30 min. The absorbance (OD) value at 615 nm was detected with a Microplate Reader (ELX-800, Biotek, Winooski, VT, USA) to determine the mass of Evans Blue dye in brain tissues per gram.

### TUNEL staining and TTC staining

Two weeks after model induction, the cell apoptotic rates in the ischemic penumbra part of brain tissues were determined with In Situ Cell Death Detection Kit (11684817910, Roche, Switzerland) according to the manufacturer’s instruction. The infarction area in the whole brain was determined with 2,3,5-triphenyltetrazolium chloride (TTC) staining. Briefly, tissue slides were incubated with 1% TTC for 10 min at 37°C: pale tissue was presumed to be infarcted. The percentage of infarcted area equal to infarcted area (mm^2^) / total area (mm^2^) × 100% was measured using the Image-Pro Plus software (Media Cybernetics, Bethesda, MD, USA).

### Immunofluorescent assay

Two weeks after model induction, ischemic penumbra parts of brain tissues were first fixed with 4% paraformaldehyde for 15 min and then incubated with 0.1% Triton X-100 for 30 min. Afterwards, the slides were blocked in 10% goat serum and incubated with primary antibodies against different indicators (Supplementary Table 1(supplementary Table 1)) overnight at 4°C. Afterwards, the cells were incubated with Cy3-labeled secondary antibody (Supplementary Table 1) for one hour in the dark, and then stained with 4,6-diamino-2-phenyl indole (DAPI) for 5 min at room temperature. Results were detected with fluorescent microscopy (BX53, OLYMPUS, Tokyo, Japan) at 400 × magnification.

### Enzyme-linked immunosorbent assay (ELISA)

Two weeks after model induction, the production of IL-6 (H007), IL-1β (H002), and TNF-α (H052) (Nanjingjiancheng Bioengineering Institute, Nanjing, Jiangsu, China) was measured using ELISA kits according to the manufacturer’s instructions.

### Reverse transcription real time polymerase chain reaction

Two weeks after model induction, total RNA in brain tissues was extracted with RNA Purified Total RNA Extraction Kit (RP1201, BioTeke, Beijing, China) and reversely transcribed into cDNA with use of Super M-MLV reverse transcriptase (RP6502, BioTeke). The reaction mixture of RT^2^-PCR system contained 10 μL of 2 × Power Taq PCR MasterMix (PR1702, BioTeke), 0.5 μL of each primer (miR-21-3p, forward: 5′- ACACTCCAGCTGGGCAACACCAGTCGATGGGC-3′, reverse: 5′- CTCAACTGGTGTCGTGGA-3′; U6, forward: 5′- CTCGCTTCGGCAGCACA-3′; reverse, 5′- AACGCTTCACGAATTTGCGT-3′), 1 μL of the cDNA template, and 8 μL of RNase-free H_2_O. Amplification was performed routinely with ExicyclerTM 96 (BIONEER, Daejeon, Republic Korea), and the relative expression level of miR-21-3p was calculated according to the formula 2^-△△ct^.

### Western blotting assay

Two weeks after model induction, total protein product was extracted with the Total Protein Extraction Kit (WLA019, Wanleibio, Shenyang, China). Then 40 μg of protein was subject to a 10% sodium dodecylsulfate polyacrylamide gel electrophoresis before membrane transfer. Primary antibodies against target proteins (Supplementary Table 1(supplementary Table 1)) were incubated with membranes at 4°C overnight, and secondary HRP IgG antibodies (1:5000) were incubated with the membranes for 45 min at 37°C. The blots were developed using Beyo ECL Plus reagent (E002-5, 7seaBiotech, Shanghai, China). The results were detected in the Gel Imaging System and analyzed with Gel-Pro-Analyzer (Media Cybernetics, Rockville, MD, USA).

### Dual luciferase assay

The direct interaction between miR-21-3p and MAT2B was detected with dual luciferase assay by Dual Luciferase Assay kit according to the manufacturer’s instruction. Wild and mutant sequences of MAT2B 3′UTR were synthesized by Sangon Biotech (Shanghai, China) and ligated to psiCHECK-2 plasmid. For luciferase reporter assay, rat normal neuron cells (0.5 × 10^4^ cells per well) (1-5065, Chi Scientific, Shanghai, China) were plated in a 96-well plate 24 hours before transfection with use of Lipofectamine 2000 according to the manufacturer’s instruction. Renilla luciferase plasmid (psi-CHECK2) was used as the internal control for the determination of transfection efficiency. Forty-eight hours after the transfection of different combinations of vectors and mimics (miR-21-3p mimics and negative control mimics, Thermo Scientific Dharmacon, Shanghai, China), fluorescence intensity was detected with a Microplate Reader (GloMax, Promega, Madison, WI, USA).

### Hypoxia/re-oxygenation model *in vitro *and miR-21-3p inhibition

Human nerve cell line SH-SY5Y (CRL-2266, ATCC) was cultured in F12 + DMEM supplemented with 10% FBS in a humidified atmosphere of 5% CO_2_ at 37°C. H/R administration of SH-SY5Y cells was performed by incubating the cells in hypoxic conditions (1% O_2_ and 5% CO_2_) at 37°C for six hours followed by 24 hours of reoxygenation. To assess the role of miR-21-3p in the ischemic neuronal injuries, miR-21-3p and negative control inhibitor (Thermo Scientific Dharmacon) were transfected into SH-SY5Y cells with Lipofectamine 2000 according to the manufacturer’s instruction. Moreover, the cells were incubated with ADMSC-derived exosomes (2 × 10^8^ particles) for 24 hours, and the effect on miR-21-3p expression was detected with RT^2^-PCR.

### Flow cytometry

The effect of miR-143-3p on cell apoptosis was assessed using an Annexin V-FITC Apoptosis Detection Kit (JingMei Biotech, Beijing, China) according to the manufacturer’s instructions, and the apoptotic rates were analyzed using a FACScan flow cytometry (Accuri C6, BD Biosciences, San Jose, CA, USA).

### Statistical analysis

Normality testing was performed with Kolmogorov-Smirnov test. The data are expressed as mean ± standard deviation (SD). The overall effect of ADMSCs was analyzed with the Kruskal-Wallis test and Wilcoxon signed rank sum test. The *P* values were adjusted using Bonferroni method. The significance level was 0.05 (two tailed *P* value). The analyses were conducted using SPSS, version 19.0 (IBM, Armonk, NY, USA).

## Results

### Identification of ADMSCs

ADMSCs were isolated from adipose tissues surrounding the epididymis of rats. ADMSCs were positive for CD29, CD40, and CD90 and negative for CD34 and CD45 (Supplementary Figure 1A(supplementary Figure 1)), which indicates typical surface antigen characteristics of ADMSCs. Compared with adipose cells, deposition of Alizarin Red (Supplementary Figure 1B(supplementary Figure 1)) and Oil Red (Supplementary Figure 1C(supplementary Figure 1)) was detected in ADMSCs, showing the cells’ osteogenic and adipogenic differentiation potential, respectively. Taken together, these results confirmed the successful generation of ADMSCs.

### ADMSCs administration improved BBB condition and suppressed apoptosis, infarction, and inflammation in brain tissues of MCAO rats

**Figure 1:**
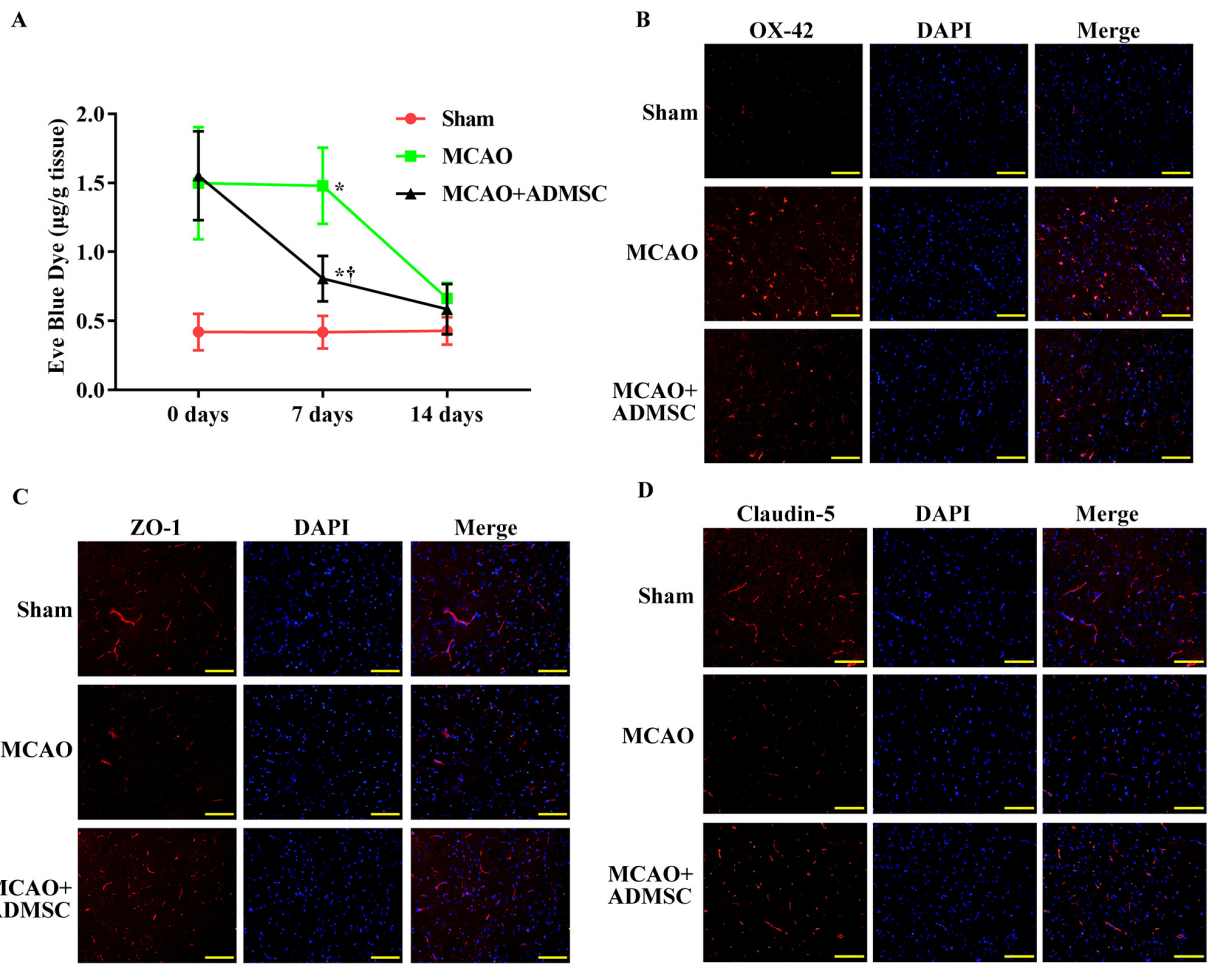
Administration of adipose-derived mesenchymal stem cells (ADMSCs) improved blood-brain barrier (BBB) condition in brain tissues of rats with middle cerebral artery occlusion (MCAO). (**A**) Quantitative analysis of Evans blue dye detection of BBB condition. (**B**) Representative images of immunofluorescence detection of OX-42. (**C**) Representative images of immunofluorescence detection of ZO-1. (**D**) Representative images of immunofluorescence detection of ZO-1. Asterisk indicates *P* < 0.05 vs sham group. Dagger indicates *P* < 0.05 vs MCAO group. Each assay was replicated six times. DAPI – 4,6-diamino-2-phenyl indole.

**Figure 2:**
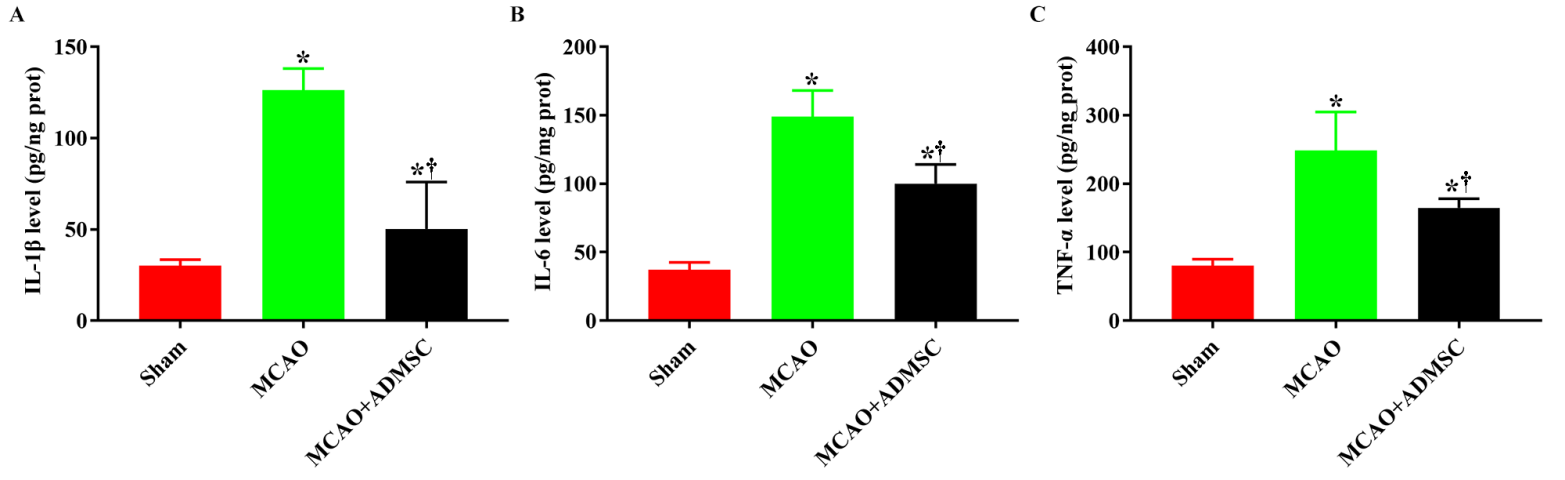
Administration of adipose-derived mesenchymal stem cells (ADMSCs) attenuated inflammation response in brain tissue of rats with middle cerebral artery occlusion (MCAO). (**A**) Quantitative analysis of IL-1β. (**B**) Quantitative analysis of IL-6. (**C**) Quantitative analysis of TNF-α. Asterisk indicates *P* < 0.05 vs sham group. Dagger indicates *P* < 0.05 vs MCAO group. Each assay was replicated six times.

**Figure 3:**
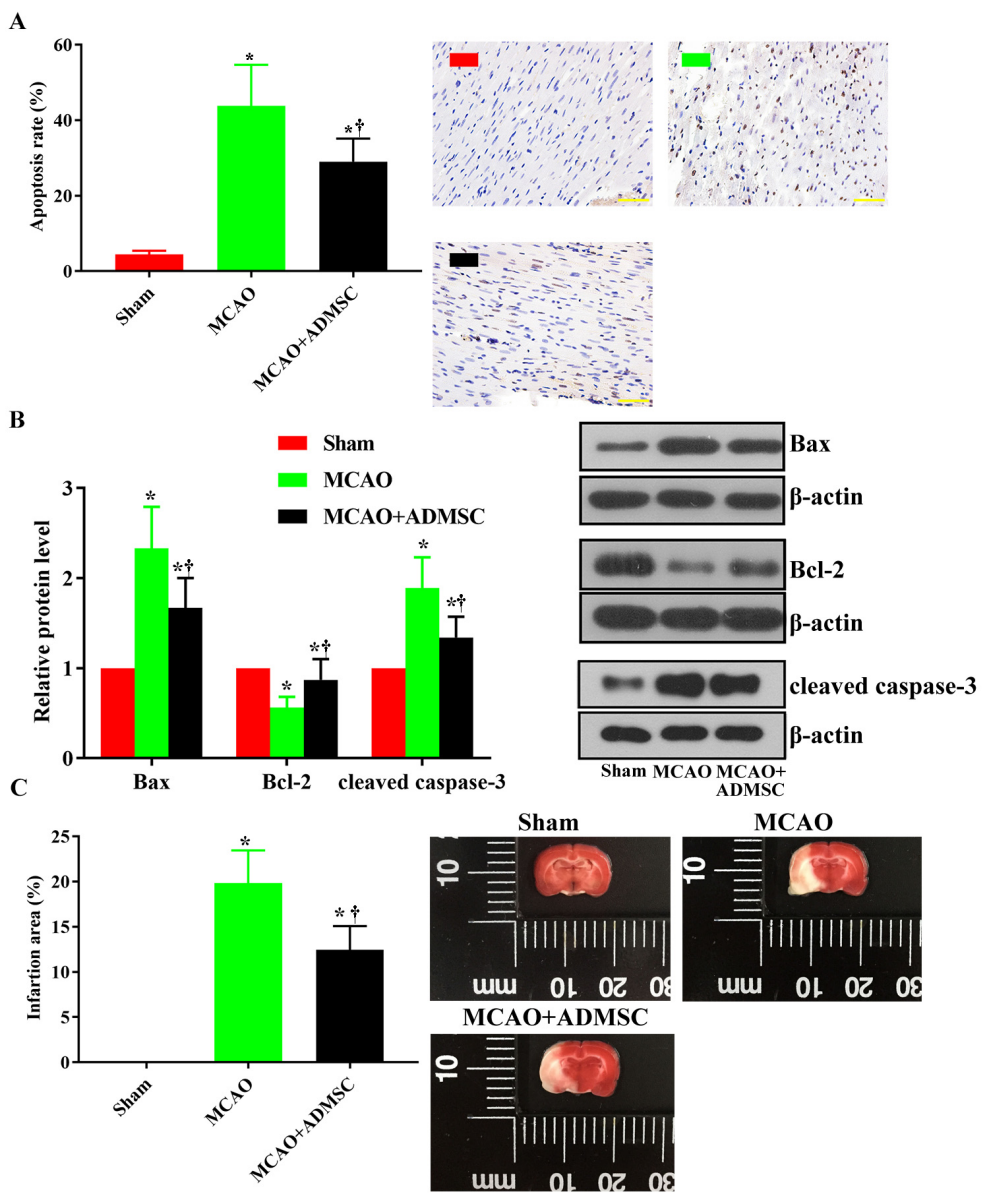
Administration of adipose-derived mesenchymal stem cells (ADMSCs) suppressed apoptosis and infarction in brain tissues of rats with middle cerebral artery occlusion (MCAO). (**A**) Quantitative analysis and representative images of TUNEL staining detection of apoptosis. (**B**) Quantitative analysis and representative images of Western blotting assays detection of Bax, Bcl-2, and cleaved caspase-3. (**C**) Quantitative analysis and representative images of 2,3,5-triphenyltetrazolium chloride staining. Asterisk indicates *P* < 0.05 vs sham group. Dagger indicates *P* < 0.05 vs MCAO group. Each assay was replicated six times.

Increased Evans Blue dye amount in rat brains indicated that ischemic injuries increased the BBB permeability. Although the BBB condition could be restored even without any treatment, the ADMSCs administration accelerated the recovery (Figure 1A). Moreover, immunofluorescent assay showed that ADMSCs administration decreased OX-42 production and distribution, and increased the distribution of ZO-1 and Claudin-5 (Figure 1B-1D), confirming the beneficial effect of ADMSCs on BBB condition. ADMSCs administration also suppressed apoptosis and inflammation and significantly inhibited the production of IL-1β, IL-6, and TNF-α when compared with the MCAO group (Figure 2A-2C) (*P* = 0.001). TUNEL assays showed that ADMSC administration decreased the proportion of apoptotic cells (stained brown) (29.3 ± 6.1%) compared with MCAO group (43.8 ± 10.9%) (*P* = 0.0001) (Figure 3). At the molecular level, ADMSCs suppressed the expression levels of Bax and cleaved caspase-3, while they increased Bcl-2 level (Figure 3; Supplementary Figure 3(supplementary Figure 3)). Moreover, TTC staining showed that the ADMSC treatment also significantly reduced the average infarction area (Figure 3C). All these results confirmed the protective effect of ADMSCs against ischemic injuries.

### Protective effect of ADMSCs on brain tissues was associated with the change in miR-21-3p-mediated MAT2B expression

**Figure 4:**
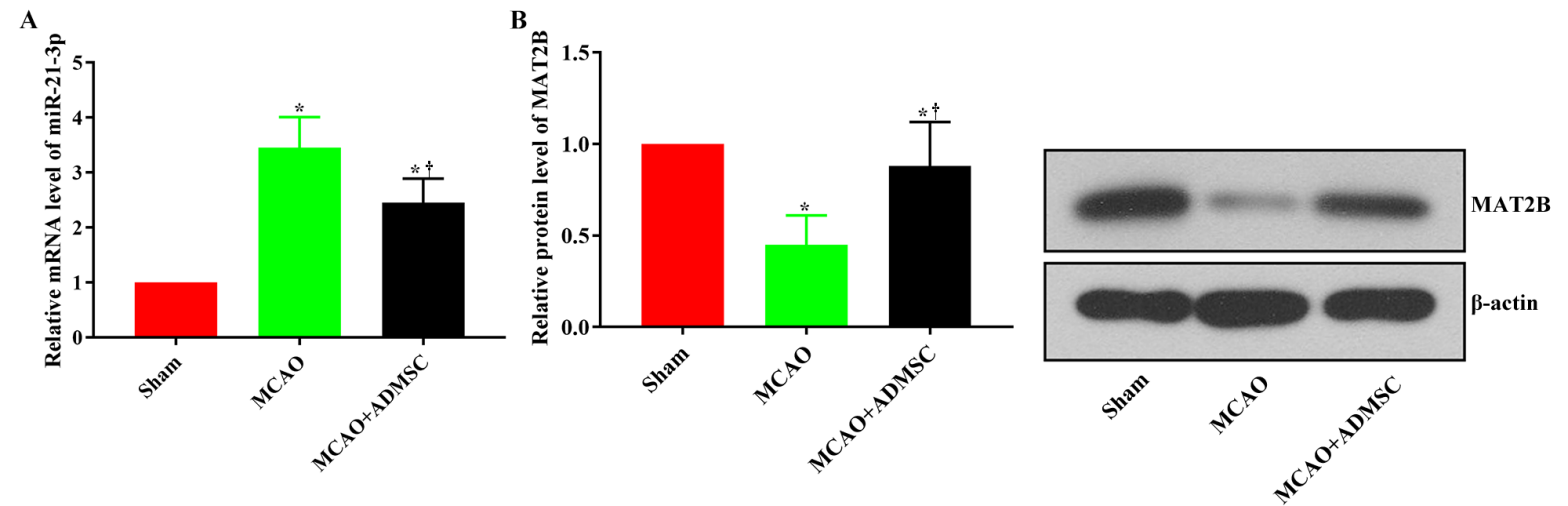
Administration of adipose-derived mesenchymal stem cells (ADMSCs) induced miR-21-3p expression and suppressed MAT2B expression in brain tissues of rats with middle cerebral artery occlusion (MCAO). (**A**) Representative analysis of reverse transcription real time polymerase chain reaction detection of miR-21-3p level. (**B**) Representative analysis and Western blotting detection of MAT2B level. Asterisk indicates *P* < 0.05 vs sham group. Dagger indicates *P* < 0.05 vs MCAO group. Each assay was replicated six times.

**Figure 5:**
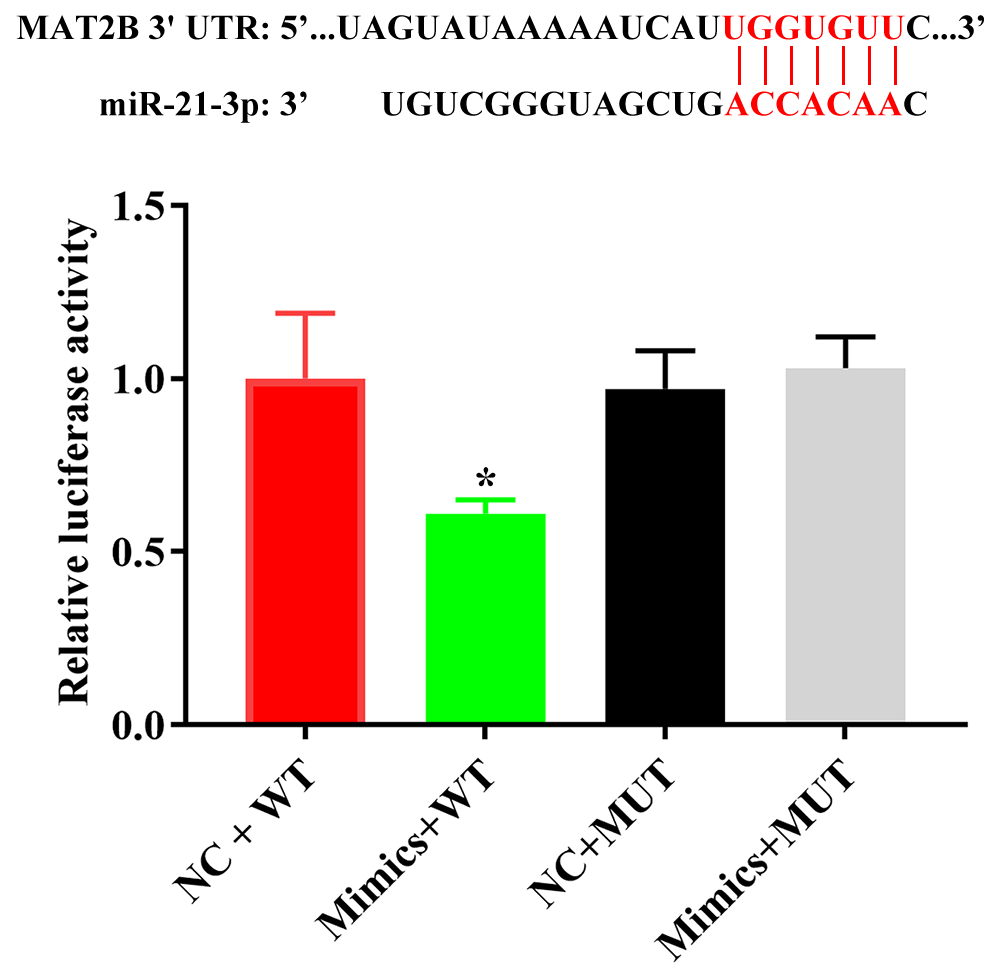
Dual luciferase assay detection of the interaction between miR-21-3p and MAT2B in normal rat neuron cells. Each assay was replicated three times. NC – negative control; WT – wild type; MUT – mutant type.

To provide more information on the treatment mechanism associated with ADMSCs, we detected the levels of miR-21-3p and its downstream effector MAT2B in rat brain tissues. The miR-21-3p level was first increased by MCAO surgery and then decreased by ADMSC administration (Figure 4A). In response to the changes in miR-21-3p level, MAT2B expression was down-regulated and then up-regulated (Figure 4B; Supplementary Figure 3(supplementary Figure 3)). To verify the direct interaction between miR-21-3p and MAT2B, we also performed dual luciferase assay with normal rat neuron cells. Co-transfection with miR-21-3p mimics and MAT2B promoter sequence suppressed luciferase activity. However, co-transfection with miR-21-3p mimics and MAT2B promoter mutant type did not influence luciferase activity (Figure 5), confirming the specific regulation effect of miR-21-3p on MAT2B in neural cells.

### Inhibition of miR-21-3p suppressed apoptosis and inflammation by inducing MAT2B expression in H/R SH-SY5Y cells

**Figure 6:**
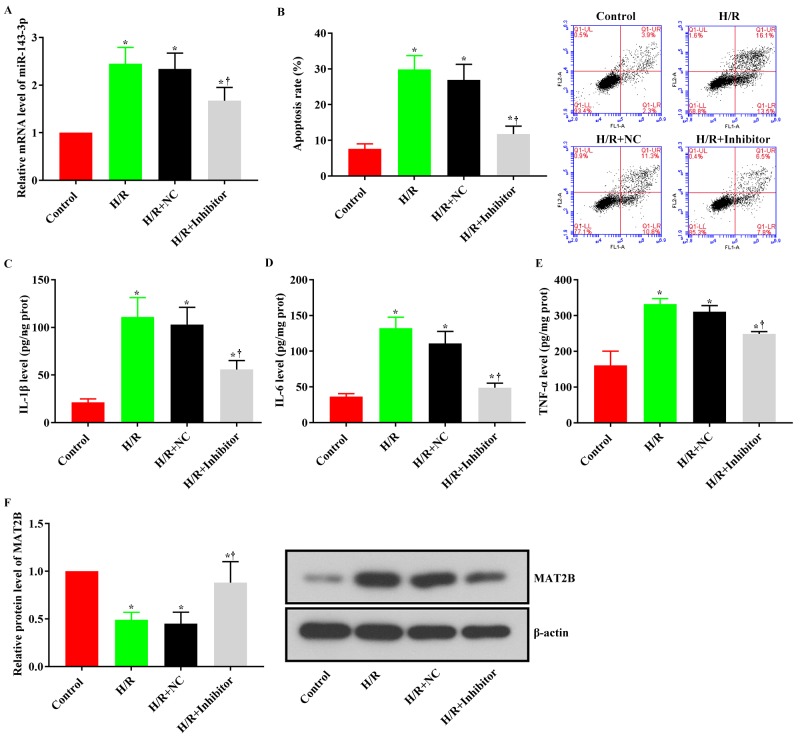
Inhibition of miR-21-3p ameliorated hypoxia/reoxygenation-induced damage in SH-SY5Y cells. **(A**) Quantitative analysis of reverse transcription real time polymerase chain reaction detection of miR-21-3p. (**B**) Quantitative analysis and representative images of flow cytometry detection of apoptosis. (**C**) Quantitative analysis results of IL-1β. (**D**) Quantitative analysis of IL-6. (**E**) Quantitative analysis of TNF-α. (**F**) Quantitative analysis and a representative image of Western blotting detection of MAT2B. Asterisk indicates *P* < 0.05 vs control group. Dagger indicates *P* < 0.05 vs H/R+Inhibitor group. Each assay was replicated six times. NC – negative control. H/R – hypoxia/re-oxygenation.

The ability of ADMSCs to protect H/R-injured neural cells by inhibiting miR-21-3p level was further verified by suppressing miR-21-3p level in human nerve cell line SH-SY5Y (Figure 6A). The increased apoptosis rate and production of IL-1β, IL-6, and TNF-α induced by H/R administration were all suppressed by miR-21-3p inhibition (Figure 6B-6E). The effect was associated with the up-regulation of MAT2B (Figure 6F; Supplementary Figure 3(supplementary Figure 3)). Moreover, the incubation of H/R SH-SY5Y cells with ADMSC-derived exosomes also decreased the miR-21-3p level (Supplementary Figure 4(supplementary Figure 4)), which supported the conclusions from the *in vivo* assays and confirmed that the inhibition of miR-21-3p by ADMSCs could serve as a promising strategy to protect brain tissues against ischemic injuries.

## Discussion

Our results showed that the treatment effect of ADMSCs on brain tissues affected by IS was associated with miR-21-3p suppression, which contributed to the MAT2B up-regulation.

MiRs are a class of small non-coding RNA members, 18 to 25 nucleotides long (8). By binding directly to the 3′-untranslated region of the targets, they regulate the level of mRNAs. miRs are also key regulators of neurodegenerative and neuropsychiatric disorders and tumors (25,26) and are involved in signaling transduction alterations-associated ischemic injuries, with either protective or detrimental effect. For example, Barteneva et al (27) reported that exosome-mediated miR-124 transfer promoted neurogenesis after ischemia. Contrary to this, Jiang et al (11) indicated that repeated remote ischemic postconditioning attenuated left ventricular remodeling via remote miR-29a transfer. In the current study, the protective effect of ADMSCs was explored by focusing on miR-21-3p function. The miR was proved to be abnormally up-regulated during IS (11,14). Moreover, Ge et al (14) attributed the impairments induced by miR-21-3p up-regulation to its suppressive effect on MAT2B. This conclusion was confirmed in our study: ADMSCs administration suppressed the miR-21-3p level *in vivo*, which up-regulated the MAT2B expression. Moreover, the dual luciferase assay with rat normal neuron cells confirmed the direct binding of miR-21-3p to the promoter of MAT2B in the nervous system, which supported the regulation sequence from miR-21-3p to MAT2B by ADMSCs. The current study also verified the possible treatment potential of miR-21-3p inhibition in ischemic injuries with human SH-SY5Y cells: the inhibition of miR-21-3p by inhibitor of ADMSC-derived exosomes suppressed apoptosis and inflammation in H/R-treated cells, which was also associated with MAT2B up-regulation.

Methionine adenosyltransferase (MAT) is an enzyme involved in cell cycle regulation (28). Mammals have three major MAT genes: MAT1A, MAT2A, and MAT2B. MAT2B encodes a regulatory β subunit, which modulates the activity of MAT2A-encoded isoenzyme (MATII) (29). The function of MAT2B is generally related to oncogenesis. For example, in hepatoma cells, TNF-induced activation of MAT2B further promoted tumor growth via NF-κB pathway (30). This interaction between MAT2B and NF-κB pathway also forms the basis of the miR-21-3p/MAT2B function in regulating inflammation (14). Thus, the effect of ADMSCs on miR-21-3p/MAT2B explained the mechanism behind anti-apoptosis and anti-inflammation effect of the cells. Moreover, the current study showed that the effect of ADMSC on miR-21-3p level was mediated indirectly via factors such as exosomes, and the inhibition factors transferred by exosomes need to be further explored.

This study confirmed the protective effect of ADMSCs on brain tissue against ischemic injury. Additionally, it also demonstrated that the effect was exerted by suppressing miR-21-3p expression, which then up-regulated MAT2B and subsequently inhibited apoptosis and inflammation in brain tissue. However, the current study is a preliminary explanation of the mechanism driving the anti-IS function of ADMSCs limited by the study approach, which failed to assess the direct effect of ADMSCs on neural cells. Moreover, we did not assess the long-term effect of ADMSCs on IS injuries. Future studies are warranted to further elucidate the mechanisms underlying the ADMSCs function.
