# Supplementary material for: Adipose-derived mesenchymal stem cells attenuate ischemic brain injuries in rats by modulating miR-21-3p/MAT2B signaling transduction
Source: Croat Med J. 2019 Oct;60(5):439–48. doi: 10.3325/cmj.2019.60.439 (PMC6852138; doi:10.3325/cmj.2019.60.439)
Supplement: Supplementary Table 1 [file CroatMedJ_60_s001.pdf]

Table S1 Detailed information of antibodies

Antibody	Product	Host species	Supplier	Country	Dilution
ZO-1	21773-1-AP	Rabbit	Proteintech	CN	1:50
Claudin-5	sc-28670	Rabbit	Santa Cruz	CN	1:50
OX-2	ab1211	Mouse	Abcam	UK	1:200
Cy3-labeled goat anti-rabbit IgG	A0516	Goat	Beyotime	CN	1:200
Cy3-labeled goat anti-mouse IgG	A0521	Goat	Beyotime	CN	1:200
MAT2B	ab109484	Rabbit	Abcam	UK	1:1000
Cleaved caspase-3	#9661	Mouse	CST	CN	1:1000
Bcl-2	bs-0032R	Rabbit	BIOSS	CN	1:500
Bax	bs-0127R	Rabbit	BIOSS	CN	1:500
Goat anti-rabbit IgG-HRP	A0208	Goat	Beyotime	CN	1:5000
Goat anti-mouse IgG-HRP	A0216	Goat	Beyotime	CN	1:5000
β-actin	sc-47778	Mouse	Santa Cruz	USA	1:1000
CD29	11-0291-80	Armenian hamster	Ebioscience	USA	1:500
CD44	12-0444-80	Mouse	Ebioscience	USA	1:1000
CD45	11-0461-80	Mouse	Ebioscience	USA	1:200
CD90	11-0900-81	Mouse	Ebioscience	USA	1:500
CD34	ab187284	Mouse	Abcam	UK	1:500
