# Supplementary figures and images for: Adipose-derived mesenchymal stem cells attenuate ischemic brain injuries in rats by modulating miR-21-3p/MAT2B signaling transduction
Source: Croat Med J. 2019 Oct;60(5):439–48. doi: 10.3325/cmj.2019.60.439 (PMC6852138; doi:10.3325/cmj.2019.60.439)

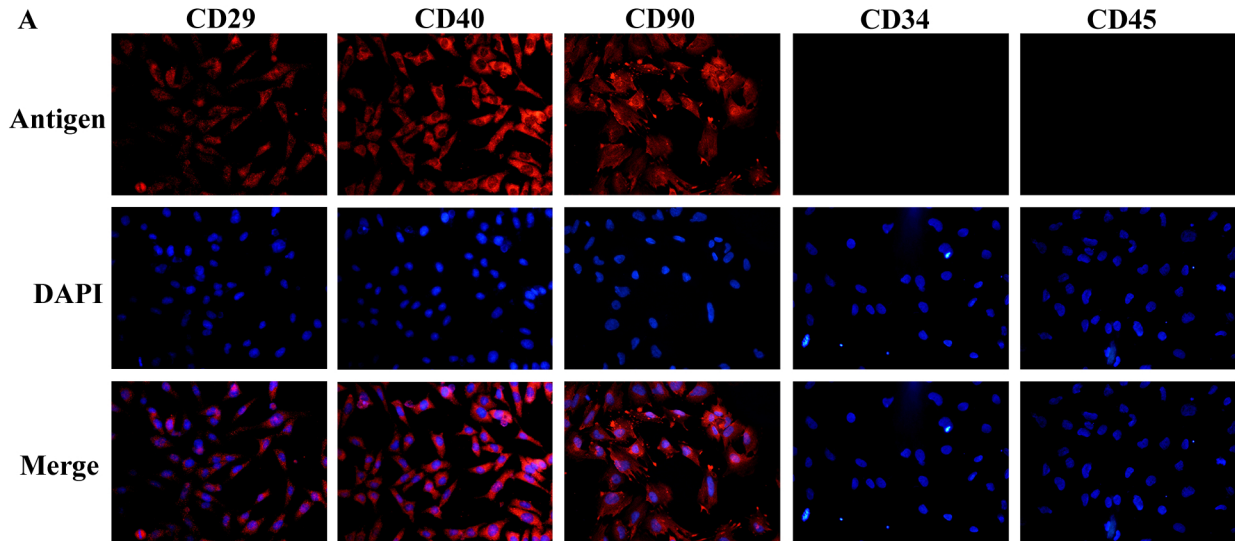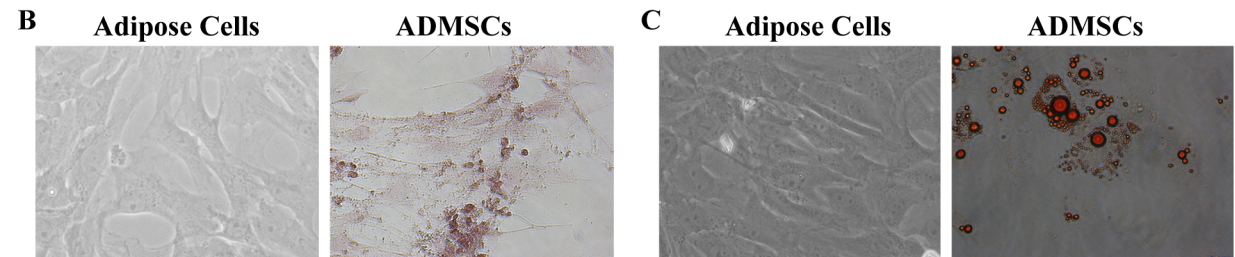

Supplement: Supplementary Figure 1 [file CroatMedJ_60_s002.pdf]

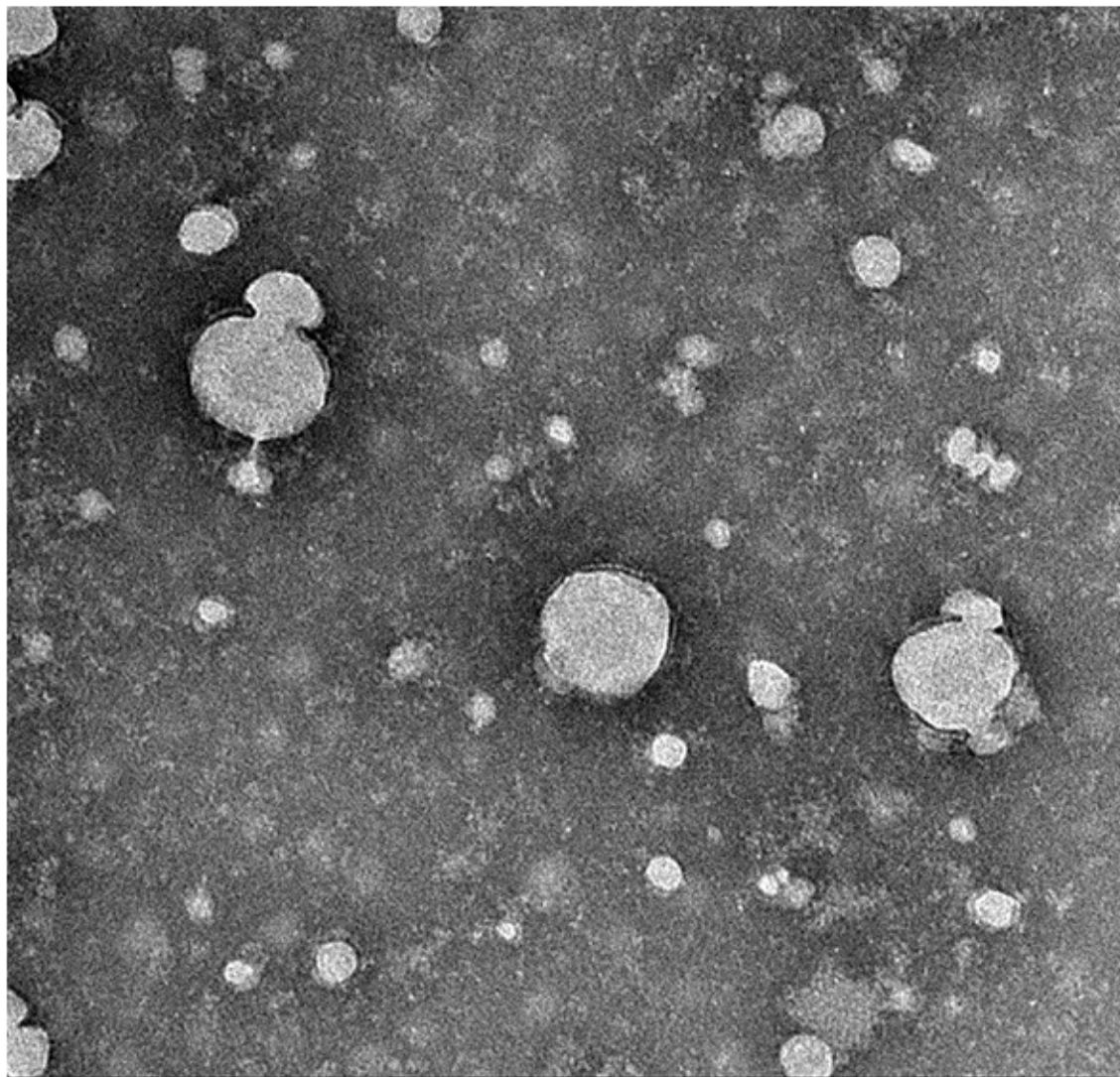

—| 100 nm

Supplement: Supplementary Figure 2 [file CroatMedJ_60_s003.pdf]

**Bax**

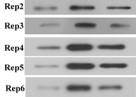

**MAT2B**

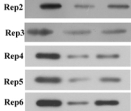

**Bcl-2**

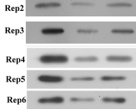

**MAT2B**

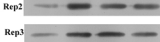

Supplement: Supplementary Figure 3 [file CroatMedJ_60_s004.pdf]

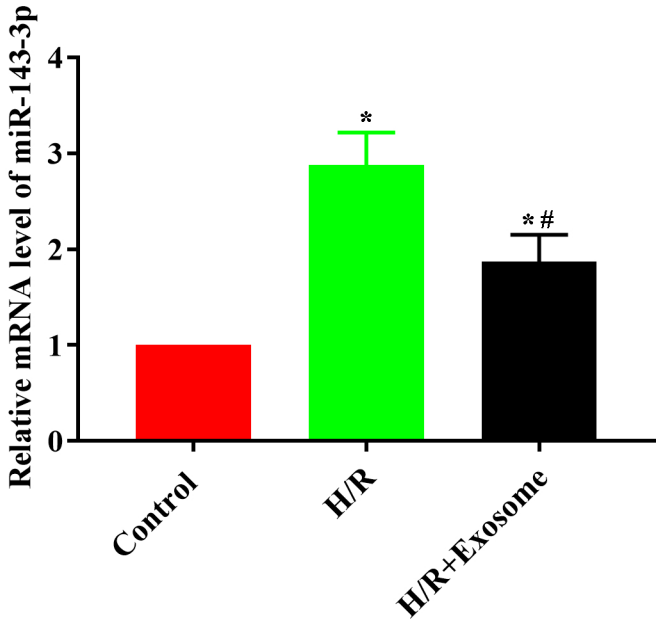

Supplement: Supplementary Figure 4 [file CroatMedJ_60_s005.pdf]
